# Quality and risk assessment of lead and cadmium in drinking water for child development centres use in Phatthalung province, Thailand

**DOI:** 10.5620/eaht.2023020

**Published:** 2023-10-12

**Authors:** Somsiri Decharat, Piriyalux Phethuayluk

**Affiliations:** 1 Department of Occupational health and Safety, Faculty of Health and Sports Science, Thaksin University, Phattalung Province 93210, Thailand; 2 Department of Public Health, Faculty of Health and Sports Science, Thaksin University, Phattalung Province 93210, Thailand

**Keywords:** Risk assessment, Lead, Cadmium, Drinking water, Child development centres

## Abstract

The purpose of this cross-sectional study and research was to evaluate the health risks to children in relation to the concentration of lead and cadmium in drinking water. Samples were collected between 1 May 2020 and 15 October 2020. Thirty-three child development centres, Phatthalung province, Thailand. Two hundred and ten drinking water samples were taken, consisting of 66 bottled water samples, 66 tap water samples, 66 filtered tap water samples and 12 raw water samples for using in the child development centres. Concentrations of lead and cadmium were identified by graphite furnace atomic absorption spectrometry. The concentration of cadmium in bottled water samples, tap water samples, filtered tap water samples, and raw water samples ranged from nd - 0.0020mg/L, nd - 0.0049 mg/L, nd - 0.0018 mg/L and nd - 0.0049 mg/L. The summation of the total hazard index of bottled water samples, tap water samples, filtered tap water, and raw water samples was less than 1, was considered health-protective. The results will provide the direct evidence needed by child development centres managers to warn learners about the health risk of drinking water among children.

## Introduction

Water is one of the 70% important components of the body, and if the body becomes dehydrated it can lead to death. Drinking clean, non-toxic water and drinking enough each day is essential [1]. However, drinking water contaminated with toxins can be harmful to health, especially contaminants in heavy metal form. Heavy metals are elements that weigh five times more than water [2]. There are both useful and toxic types of heavy metals in the body, and eating is the most important channel that carries these substances into the body [3]. These substances may contaminate drinking water during the production process, and heavy metals, such as lead, cadmium, mercury, iron, manganese, copper, etc. are often found in contaminated drinking water. The consumption of water containing these heavy metals can affect health [1],[3],[4],[5].

Early childhood is an important stage of development and an important foundation for the development of the next generation. A good early childhood development facility must have the appropriate environmental health management. If this is lacking, it may be lead to health problems related to environmental factors, such as accidents, gastrointestinal diseases, dengue fever, hand, foot and mouth disease, etc., [6], [7]. Therefore, monitoring the quality of drinking water in child development centres is essential, as exposure to toxic metals in contaminated drinking water can affect the health of schoolchildren. Lead exposure is especially dangerous to children’s developing brains and can result in reduced intelligence quotient (IQ), attention span, impaired learning ability, and increased risk of behavioural problems [3]. In addition, exposure to cadmium in childhood may be associated with the development of various diseases in children or later life [8], [9].

Children exposed to mercury may show redness of the cheeks, nose, and lips; loss of hair, teeth, and nails; transient rashes; hypotonia (muscle weakness); and increased sensitivity to light. Other symptoms may include kidney dysfunction (e.g. Fanconi syndrome) or neuropsychiatric symptoms such as emotional lability, memory impairment, or insomnia [10]. Many studies have shown drinking water contaminated by heavy metals. Ahmed J et al., [11] reported on 425 sampled schools among primary school children of Pakistan, whose levels of heavy metals in the drinking water often exceeded the WHO permissible limits. They found the schools exceeded the limit of Pb (67%), Cd (17%), and Fe (15%), and their findings, particularly for Pb, are of concern, as Pb may negatively influence children’s growth, development, school performance, and long-term health. Liqing L et al., [12] reported that the risk posed by non-carcinogens due to drinking water contaminated with heavy metals (Pb, Cd, As) was less than 1. The range of carcinogenic risks related directly to drinking water was within the range of 1.07×10-8/year to 5.58×10-6/year, which is within the acceptable level. Guidelines for drinking-water quality (GDWQ) [13] include the following recommended limits on naturally occurring constituents that may have direct adverse health impacts: lead (Pb) 0.01 parts per million (ppm.) and cadmium (Cd) 0.003 ppm. In addition, the Environmental Protection Agency recommendation is lead 0.015 ppm., and Cd 0.005 ppm [14]. In this study, the researchers chose lead and cadmium among the various heavy metals because children are at higher risk of lead poisoning; when they come into contact with environmental lead, the severity of poisoning increases [15]. Lead can enter drinking water when plumbing materials that contain lead corrode [16]. Additionally, seepage from hazardous waste sites as well as mining or industrial effluent can pollute areas with high levels of cadmium. Lead and cadmium are frequently removed from water lines by soft or acidic water, and the levels of cadmium in stagnant water inside of domestic pipes rise [15].

The primary sources of drinking water in child development centres are bottled water, tap water and filtered water. Surface water is used as potable water in some schools and raw water is also used to clean containers, vegetables, etc. Lead and cadmium concentrations as well as health risks associated with exposure to these heavy metals should be examined. The objectives of this study were to study the concentration of lead and cadmium in drinking water samples and to evaluate the health risk from exposure to lead and cadmium in drinking water used in child development centres.

## Materials and Methods

This cross-sectional study was conducted between 1 May 2020 and 15 October 2020 in child development centres located in four subdistricts, including the Papayom, Koh Tao, Lan Khoi, and Ban Phrao in the Papayom districts, and two subdistricts including Kong Ra and Khlong Chalerm in the Phatthalung province, Thailand, which are supported by local government organizations. Thirty-three child development centres were selected using a purposive sampling method. A total of 210 drinking water samples were collected, including 66 bottled water samples, 66 tap water samples, 66 filtered tap water samples and 12 raw water samples.

### Sample collection, sample preparation, measuring, and analysis

Most potable water samples were taken directly from the tap or source after letting the water run for at least 5 minutes. Water samples were stored in 50 polyethylene bottles previously washed in nitric acid. The water samples were acidified with 50% (v/v) nitric acid (E. Merck, Darmstadt, Germany) to bring the pH down to below 2 and tested within two days. All samples were filtered before they were analysed using Whatman® Ashless Filters, Grade 541, (Whatman, London, UK) that minimize suspended solids associated with the risk of capillary tube obstruction in the instrument.

Water samples were tested for pH determination with the pH-meter instrument (Eutech Instruments, Cyber Scan pH 510), and measured turbidity in drinking water with a turbidity meter (HANNA, HI98703 Tungsten light source).

In order to create the standards and dilution standards for analyses, stock solutions of Pb and Cd (1000 mg/L) were diluted using 1.0 mol/L of nitric acid. The calibration solutions and 20 μL of samples were pipetted into the graphite tube for measurement. Concentrations of lead and cadmium were identified using graphite furnace atomic absorption spectrometry (GFAAS, PerkinElmer, Analyst 800). Standards and dilution standards for assays followed US, EPA, and Health Canada requirements [17]. The standard reference material (trace element in water) was used to assess the methods used for the measurement of lead and cadmium in drinking water samples. All samples were analysed in triplicate. GFASS element detection limits were 0.003 mg/L for lead and 0.0002 mg/L for cadmium.

### Risk assessment

The United States Environmental Protection Agency test method (US. EPA) [18] was used to calculate the intake from accidental ingestion of lead and cadmium in drinking water as follows:


(1) Intake (mg/kg × day) = C × IR × EF × ED/BW × AT

where C is the concentration of lead and cadmium in drinking water (mg/L) (data from laboratory analysis), IR is the ingestion rate (1.950 L/day for age less than 6 years old [18], EF is the exposure frequency (360 days/year)[16], ED is the exposure duration (6 years) [19], BW is the body weight (average of body weight of female students 16.60 kg; n = 585 students; max = 21.7 kg, min = 11.50 kg; average of body weight of male students 17.7 kg; n = 412 students; max = 22.6 kg, min = 12.10 kg ) (data from questionnaires), AT is the average time of exposure (ED×365 days/year)[19]. When Hazard quotient (HQ) and hazard index (HI) values are below one, there is no risk to the population, but if these values are above one, potential carcinogenic effects may be a concern [18].

A hazard quotient (HQ) is the ratio of the potential exposure to a selected metal, relative to the level at which no adverse effects are expected [16]. HQ can be obtained using Equations (2) and (3):


(2) HQ = Dose intake/RfD

RfD is the reference dose (mg kg−1 day−1). In addition, Hazard Index (HI) is calculated by summing the individual HQs to assess the total health risks of all selected target metals. If the calculated HQ is < 1, then no adverse health effects are expected because of exposure. Conversely, if HQ is >1, then negative health impacts are possible [16].


(3) HI = Σ HQi

For calculate risk of samples where lead and cadmium concentrations cannot be measured. We use limit of detection (LOD) instead of the value to calculate the risk equation.

### Data analysis

Descriptive statistics were used to assess the concentrations of lead and cadmium in drinking water in the child development centres.

## Results and Discussion

Drinking water samples were collected for analysis from 33 child development centres. Temperature, pH and turbidity were measured following the American Public Health Association [18]. The temperature, pH and turbidity of bottled water samples (66 samples) were shown as 25.5–26.3 °C, pH 6.0–6.5, and without turbidity respectively. For the temperature, pH and turbidity of tap water samples (66 samples) were shown as 25.0 –25.5 °C, pH 6.5–6.8 and 1.3–1.8 Nephelometric Turbidity Units (NTU), respectively. In addition, the temperature, pH and turbidity of filtered tap water samples (66 samples) and raw samples were shown as 25.5–25.8 °C, pH 6.0–6.5, not detected and 25.3–26.3 °C, pH 6.0–6.3 and 3.2–3.5 NTU, respectively (Table 1).

The concentration of lead in bottled water samples, tap water samples, filtered tap water samples and raw water samples ranged from <0.0003 to 0.0162 mg/L, <0.0003 to 0.0260 mg/L, <0.0003 to 0.0170 mg/L, and <0.0003 to 0.0400 mg/L, respectively. In addition, the concentration of cadmium in bottled water samples, tap water samples, filtered tap water samples and raw water samples ranged from <0.0002 to 0.0020 mg/L, <0.0002 to 0.0049 mg/L, <0.0002 to 0.0018 mg/L and <0.0002 to 0.0049 mg/L, respectively (Table 1).

### Concentration of lead and cadmium in drink water samples, and risk assessment

The concentration of Pb in the bottled water intake among the female students was 0.0008 mg/kg, and among the male students 0.0003 mg/kg. In addition, intake among the female students was 0.0008 mg/kg, and among the males 0.0007 mg/kg. The concentration of Pb in the tap water intake among the female students was 0.0002 mg/kg, and this was the same among the males (0.0002 mg/kg). In addition, intake among both sets of students was 0.0006 mg/kg. The concentration of Pb in filtered tap water intake was also the same in both groups at 0.0002 mg/kg. In addition, the intake among both sets/groups of students was 0.0004 mg/kg. The concentration of Pb in the raw water intake among the female students was 0.0026 mg/kg, and in the males, it was 0.0024 mg/kg. In addition, intake among both sets of students was 0.0002 mg/kg. The hazard quotients of Pb and Cd were less than 1 in all water samples and the hazard quotients of Pb and Cd were less than 1 (Table 2).

The temperature, pH and turbidity results for all samples in this study did not exceed the Thai Ministry of Public Health [21] and WHO [22] recommended allowable limits. The level of lead found in 8.34% of the bottled water samples exceeded the recommended authorized limit for lead set by both Thailand Ministry of Public Health [21] and WHO [22] recommendations for drinking water quality. However, the concentration of cadmium in the bottled water samples did not exceed the permissible level of cadmium for the drinking water quality standard established by both Thailand Ministry of Public Health [21] and WHO [22]. Reverse osmosis (RO), UV treatment, ozone treatment, and mineral or spring water are the production processes of bottled water in Thailand that will affect the price of drinking water. However, some of the bottled water brands in this study were produced in a local community factory, and the production process has not yet been standardized, has not yet been standardized – for example, lead was found in some of the brands. Almost one-tenth (9.09%) of bottled water samples (range 0.0140–0.0162 mg/L), exceeded the permissible values of lead and cadmium set by both Thailand Ministry of Public Health [21] and WHO [22] in their guidelines for drinking water quality. However, the big bottle water companies in Thailand have large water purification facilities and are more likely to adhere to a good standardized manufacturing process.

Most tap water samples did not exceed the allowable values for lead and cadmium, according to the drinking water quality guidelines established by Thailand Ministry of Public Health [21] and WHO [22]. However, the lead and cadmium concentrations in 12.12% (ranging from 0.0190to 0.0260 mg/L) and 12.12% (ranging from 0.0040to 0.0049 mg/L) of tap water samples exceeded the permissible values of lead and cadmium set by those guidelines.

Some studies have documented the dissolution of lead in tap water when lead-free taps were used, which has been attributed to brass fittings in domestic plumbing systems. Thus, it is suggested that faucets made from a material that does not contain lead (unleaded faucets) should be used, such as stainless-steel faucets. Also, rinsing the faucets before consuming water from them for two or three minutes is a technique that can help reduce the concentration of contaminating heavy metals [23], [24], [25].

The production process of tap water commonly uses chlorine for disinfection. It has been suggested that the presence of chlorine and other pollutants in tap water, such as trihalomethanes (THMs), may lead to the development of a common carcinogen, chloroform. Hadi Sadeghi et al., [26] showed in their results that the concentrations of THMs were higher in summer than in other seasons as well as the mean values of lifetime cancer risks for residents through ingestion, inhalation, and dermal contact. Emilie Helte et al., [27] reported that there is no overall association of THM with the risk of bladder cancer, hazard ratio for the highest exposed compared to the non-exposed 0.90 (95% confidence interval: 0.73–1.11).

Most filtered tap water samples did not exceed the allowable values for lead and cadmium, according to the drinking water quality guidelines established by Thailand and the WHO. However, the lead concentrations in 12.12% of the tap water samples (ranging from 0.019 to 0.0260 mg/L), exceeded the permissible values of lead and cadmium set by both the Thailand Ministry of Public Health [21] and WHO [22] guidelines for drinking water quality that drinking water standards for lead=0.01 mg/L, and cadmium=0.003 mg/L. Traditional water filtration methods are not available and offer many other options, such as boiling, UV treatment, disinfectant tablets, portable sediment filters and charcoal. In addition, the dual function of the filtration and adsorption membrane is highly effective in removing minute quantities of pollutants, such as cationic heavy metals. Nguyen TT et al., [28] reported that in Cd removal tests, conducted in both batch and continuous adsorption experiments, the maximum treatment efficiency and adsorption capacity were determined to be 99.97% and 10 mg/g of material, respectively. Ethaib S et al., [29] reported on the various types of nanoparticle-structured materials that showed a high removal efficiency for different types of heavy metals with excellent adsorption capacity and selectivity such as carbonaceous, zeolite, polymer-based, magnetic and metal oxide, carbon materials, and magnetic and silica-based materials.

The concentration of lead and cadmium in 33.34% (range 0.0180–0.0400 mg/L) and 16.67% (range 0.0031–0.0049 mg/L) of raw water samples, respectively, exceeded the recommended permissible limit set by Thailand and WHO in their guidelines for drinking water quality. An area’s quality of surface water is highly affected by human activities in that area [30]. In this study, most of the farming activities are responsible for water pollution due to excessive use of pesticides and chemical fertilizers, which may have a profound effect on the surface water [31], [32], [33] because most of the southern region's farmers use open field farming in which the agricultural zones, para-rubber, rice and garden fruits, are located near the surface water source. Agricultural activities may be linked to surface contamination from pesticides, heavy meals, various chemicals and other contaminants. Meanwhile, some child development centres are close to livestock farms. Water sources are also vulnerable to bacterial contamination. As a result, raw water samples are unsuitable for consumption unless they are processed by water management organizations.

The result of the hazards index (HI) from drinking waters in this study was less than 1, suggesting there was considered health-protective. However, Children are growing and are sensitive to toxins. Therefore, surveillance of heavy metal contamination in drinking water is essential. Based on several studies, postnatal blood lead (PbB) concentrations are negatively associated with child growth [34, 35, 36]. Renzetti et al. [37] and Dallaire et al. [38] reported that increased maternal PbB levels are associated with preterm birth, low birth weight, and lower height and weight gains in early childhood and childhood. In addition, low cadmium exposure is still limited and has opposing results. Lalit et al. [39] reported that even moderate or high exposure to this metal during pregnancy could have serious health consequences, which could be reflected in children’s early or later stages of life. Kim W et al. [40] reported that increased prenatal Cd concentrations were associated with increased scores for attention deficit hyperactivity disorder (ADHD) for girls, but not for boys, at the age of six.

## Conclusions

The levels of lead and cadmium found in filtered tap water did not exceed the allowable limits recommended by Thailand and the WHO. The findings provide direct evidence that school managers need to inform learners of the health risk of children’s exposure to the ingestion of high levels of heavy metal ingestion. Thus, the production of bottled water in local water facilities should be monitored to ensure that facilities have the appropriate technology to meet drinking water standards. In addition, raw water is unsuitable for consumption unless it is processed by water agencies.

## Figures and Tables

**Table 1.** The physical and chemical properties of drinking water use in the child development centers (n = 210)

Sources	Temperature Range (°C)	pH Range	Turbidity Range (NTU)	Lead concentrations ^a^ (mg/L)		Cadmium concentrations ^1^ (mg/L)	
				n ≤ 0.01 mg/L (%) (range mg/L)	n > 0.01 mg/L (%) (range mg/L)	n ≤ 0.003 mg/L (%) (range mg/L)	n > 0.003 mg/L (%) (range mg/L)
Bottled water (n = 66)	25.5 -26.3	6.0 -6.5	without turbidity	60 (90.91) (<0.0003*–0.0032)	6 (9.09) (0.014–0.0162)	66 (100.00) (<0.0002*–0.0020)	0
Tap water (n = 66)	25.0 -25.5	6.5 -6.8	1.3-1.8	58 (87.88) (<0.0003*–0.0028)	8 (12.12) (0.019–0.0260)	58 (87.88) (<0.0002*–0.0012)	8 (12.12) (0.0040–0.0049)
Filtered tap water (n = 66)	25.5 -25.8	6.0 -6.5	without turbidity	62 (93.94) (<0.0003*– 0.007)	4 (6.06) (0.012-0.017)	66 (100.00) (<0.0002*–0.0018)	0
Raw water (n = 12)	25.3 -26.3	6.0 -6.3	3.2-3.5	6 (66.66) (<0.0003*–0.0049)	6 (33.34) (0.0180–0.0400)	20 (83.33) (<0.0002*–0.0024)	4 (16.67) (0.0031–0.0049)

1 WHO guide limit level. LOD = limit of detection.

LOD for Lead = 0.0003 mg/L. LOD for cadmium = 0.0002 mg/L The results were *less than the limit of quantification of the method (LOQ).

**Table 2.** Chronic daily intake (CDI), Hazard Quotient (HQ) and Hazard Index (HI) in children due to lead and cadmium from different drinking water sources (n = 210)

Sources	Intake from average drinking water ingestion of Pb (Female student)	Intake from average drinking water ingestion of Pb (Male students)	Intake from average drinking water ingestion of Cd (Female students)	Intake from average drinking water ingestion of Cd (Male students)	HQ value average of Pb (Female students)	HQ value average of Pb (Male students)	HQ value average of Cd (Female students)	HQ value average of Cd (Male students)	HI
Bottled water (n = 66)	0.0008	0.0003	0.0008	0.0007	0.0800	0.0300	0.2667	0.2333	0.1525
Tap water (n = 66)	0.0002	0.0002	0.0006	0.0006	0.0200	0.0200	0.2000	0.2000	0.1100
Filtered tap water (n = 66)	0.0002	0.0002	0.0004	0.0004	0.0200	0.0200	0.1333	0.1333	0.0767
Raw water (n = 12)	0.0026	0.0024	0.0002	0.0002	0.0260	0.0240	0.0667	0.0667	

Drinking water standards for lead=0.01 mg/L, and cadmium=0.003 mg/L [14].

## References

[1] https://www.who.int/newsroom/fact-sheets/detail/lead-poisoning-and-health.

[2] Banfalvi G (2011). Cellular Effects of heavy metals.

[3] https://www.cdc.gov/niosh/topics/lead/workerinfo.html.

[4] Jaishankar M, Tseten T, Anbalagan N, Mathew BB, Beeregowda KN (2014). Toxicity, mechanism and health effects of some heavy metals. Interdiscip Toxicol.

[5] Spiller HA (2018). Rethinking mercury: the role of selenium in the pathophysiology of mercury toxicity. Clin Toxicol (Phila).

[6] https://apps.hpc.go.th/dl/web/upFile/2021/12-4052-20211206130901/ddd93393299b182eca644ed37997295f.pdf.

[7] https://www.cdc.gov/nceh/lead/prevention/health-effects.htm.

[8] Dursun A, Yurdakok K, Yalcin SS, Tekinalp G, Aykut O, Orhan G (2016). Maternal risk factors associated with lead, mercury and cadmium levels in umbilical cord blood, breast milk and newborn hair. J Matern Fetal Neonatal Med.

[9] Luo Y, McCullough LE, Tzeng JY, Darrah T, Vengosh A, Maguire RL (2017). Maternal blood cadmium, lead and arsenic levels, nutrient combinations, and offspring birthweight. BMC Public Health.

[10] Johnson-Arbor K, Tefera E, Farrell Jr J (2021). Characteristics and treatment of elemental mercury intoxication: A case series. Health Sci Rep.

[11] Ahmed J, Wong LP, Chua YP, Channa N, Memon UUR, Garn JV (2021). Heavy metals drinking water contamination and health risk assessment among primary school children of Pakistan. J Environ Sci Health A Tox Hazard Subst Environ Eng.

[12] Li L, Huang Y, Liu Y (2021). Health risk assessment of heavy metals in direct drinking water among middle and primary schools in Huangpi District of Wuhan[J]. Chinese Journal of School Health.

[13] https://www.who.int/publications/i/item/9789241549950.

[14] https://www.epa.gov/ground-water-and-drinking-water/national-primary-drinking-water-regulations.

[15] Loh N, Loh HP, Wang LK, Wang M (2016). Health effects and control of toxic lead in the environment. Handbook of Environmental Engineering.

[16] https://www.cdc.gov/nceh/lead/default.htm.

[17] https://soctrade.ua/upload/broshury/6Trace_Metals_Waters_GFAAS.pdf.

[18] https://www.epa.gov/sites/default/files/2015-09/documents/rags_a.pdf.

[19] https://www.who.int/tools/growth-reference-data-for-5to19-years.

[20] American Public Health Association (APHA) (1998). Standard methods for the examination of water and wastewater.

[21] https://www.fao.org/faolex/results/details/en/c/LEX-FAOC164413/.

[22] https://www.epd.gov.hk/eia/register/report/eiareport/eia_2242014/EIA/app/app02.02.pdf.

[23] Harvey PJ, Handley HK, Taylor MP (2016). Widespread copper and lead contamination of household drinking water, New South Wales, Australia. Environ Res.

[24] Ng DQ, Lin YP (2016). Evaluation of lead release in a simulated lead-free premise plumbing system using a sequential sampling approach. Int J Environ Res Public Health.

[25] https://www.health.gov.au/sites/default/files/documents/2020/02/enhealth-guidance-lead-in-drinking-waterfrom-some-plumbing-products-enhealth-guidance-lead-in-drinking-water-from-some-plumbing-products.pdf.

[26] Sadeghi H, Nasseri S, Yunesian M, Mahvi AH, Nabizadeh R, Alimohammadi M (2019). Trihalomethanes in urban drinking water: measuring exposures and assessing carcinogenic risk. J Environ Health Sci Eng.

[27] Helte E, Säve-Söderbergh M, Ugge H, Fall K, Larsson SC, Å kesson A (2022). Chlorination by-products in drinking water and risk of bladder cancer – A population-based cohort study. Water Research.

[28] Nguyen TT, Thi TMLP, Thi TN, Le TT, Thi CTN, Nguyen NH (2023). Adsorption optimization for the removal of cadmium in water by aluminum (Hydr)oxide on cation exchange resin. Current Applied Science and Technology.

[29] Ethaib S, Al-Qutaifia S, Al-Ansari N, Zubaidi SL (2022). Function of nanomaterials in removing heavy metals for water and wastewater remediation:A review. Environments.

[30] https://www.who.int/publications/i/item/9241544805.

[31] Naveen Kumar N, Kumar A, Marwein BM, Verma DK, Jayabalan I, Kumar A (2021). Agricultural activities causing water pollution and its mitigation – A review. International Journal of Modern Agriculture.

[32] Rad SM, Ray AK, Barghi S (2022). Water pollution and agriculture pesticide. Clean Technologies.

[33] Shen Y, Zhao E, Zhang W, Baccarelli AA, Gao F (2022). Predicting pesticide dissipation half-life intervals in plants with machine learning models. J Hazard Mater.

[34] Deierlein AL, Teitelbaum SL, Windham GC, Pinney SM, Galvez MP, Caldwell KL (2019). Lead exposure during childhood and subsequent anthropometry through adolescence in girls. Environ Int.

[35] Tatsuta N, Nakai K, Kasanuma Y, Iwai-Shimada M, Sakamoto M, Murata K (2020). Prenatal and postnatal lead exposures and intellectual development among 12-year-old Japanese children. Environ Res.

[36] Wang M, Xia W, Zeng Q, Zhang W, Qian X, Bao S (2021). Associations between prenatal and postnatal lead exposure and preschool children humoral and cellular immune responses. Ecotoxicol Environ Saf.

[37] Renzetti S, Just AC, Burris HH, Oken E, Amarasiriwardena C, Svensson K (2017). The association of lead exposure during pregnancy and childhood anthropometry in the Mexican PROGRESS cohort. Environ Res.

[38] Dallaire R, Dewailly E, Ayotte P, Forget-Dubois N, Jacobson SW, Jacobson JL (2014). Growth in Inuit children exposed to polychlorinated biphenyls and lead during fetal development and childhood. Environ Res.

[39] Chandravanshi L, Shiv K, Kumar S (2021). Developmental toxicity of cadmium in infants and children: a review. Environ Anal Health Toxicol.

[40] Kim W, Jang Y, Lim YH, Kim BN, Shin CH, Lee YA (2020). The effect of prenatal cadmium exposure on attention-deficit/hyperactivity disorder in 6-year-old children in Korea. J Prev Med Public Health.

